# Molecular Processes Studied at a Single-Molecule Level Using DNA Origami Nanostructures and Atomic Force Microscopy

**DOI:** 10.3390/molecules190913803

**Published:** 2014-09-03

**Authors:** Ilko Bald, Adrian Keller

**Affiliations:** 1 Institute of Chemistry—Physical Chemistry, Universität Potsdam, Karl-Liebknecht-Straße 24-25, D-14476 Potsdam, Germany; 2 BAM Federal Institute of Materials Research and Testing, Richard-Willstätter Str. 11, D-12489 Berlin, Germany; 3 Technical and Macromolecular Chemistry, University of Paderborn, Warburger Str. 100, D-33098 Paderborn, Germany

**Keywords:** DNA origami, atomic force microscopy, single-molecule analysis, DNA radiation damage, protein binding, enzyme reactions, G quadruplexes

## Abstract

DNA origami nanostructures allow for the arrangement of different functionalities such as proteins, specific DNA structures, nanoparticles, and various chemical modifications with unprecedented precision. The arranged functional entities can be visualized by atomic force microscopy (AFM) which enables the study of molecular processes at a single-molecular level. Examples comprise the investigation of chemical reactions, electron-induced bond breaking, enzymatic binding and cleavage events, and conformational transitions in DNA. In this paper, we provide an overview of the advances achieved in the field of single-molecule investigations by applying atomic force microscopy to functionalized DNA origami substrates.

## 1. Introduction

During the last three decades, the field of structural DNA nanotechnology has developed a variety of techniques to assemble DNA into increasingly complex nanostructures [1]. The unique self-assembly capabilities of DNA result from the strong specificity of Watson-Crick base pairing. By controlling the nucleobase sequence of DNA strands, different segments along a given strand can be programmed to pair with different partners, thus enabling the formation of branched junctions. These junctions can be used as building blocks and further assembled into larger arrangements, albeit only with rather moderate assembly yields [2]. However, with the introduction of the DNA origami technique by Rothemund in 2006 [3], the rapid, high-yield assembly of complex, well-defined DNA nanostructures suddenly became feasible.

**Figure 1:**
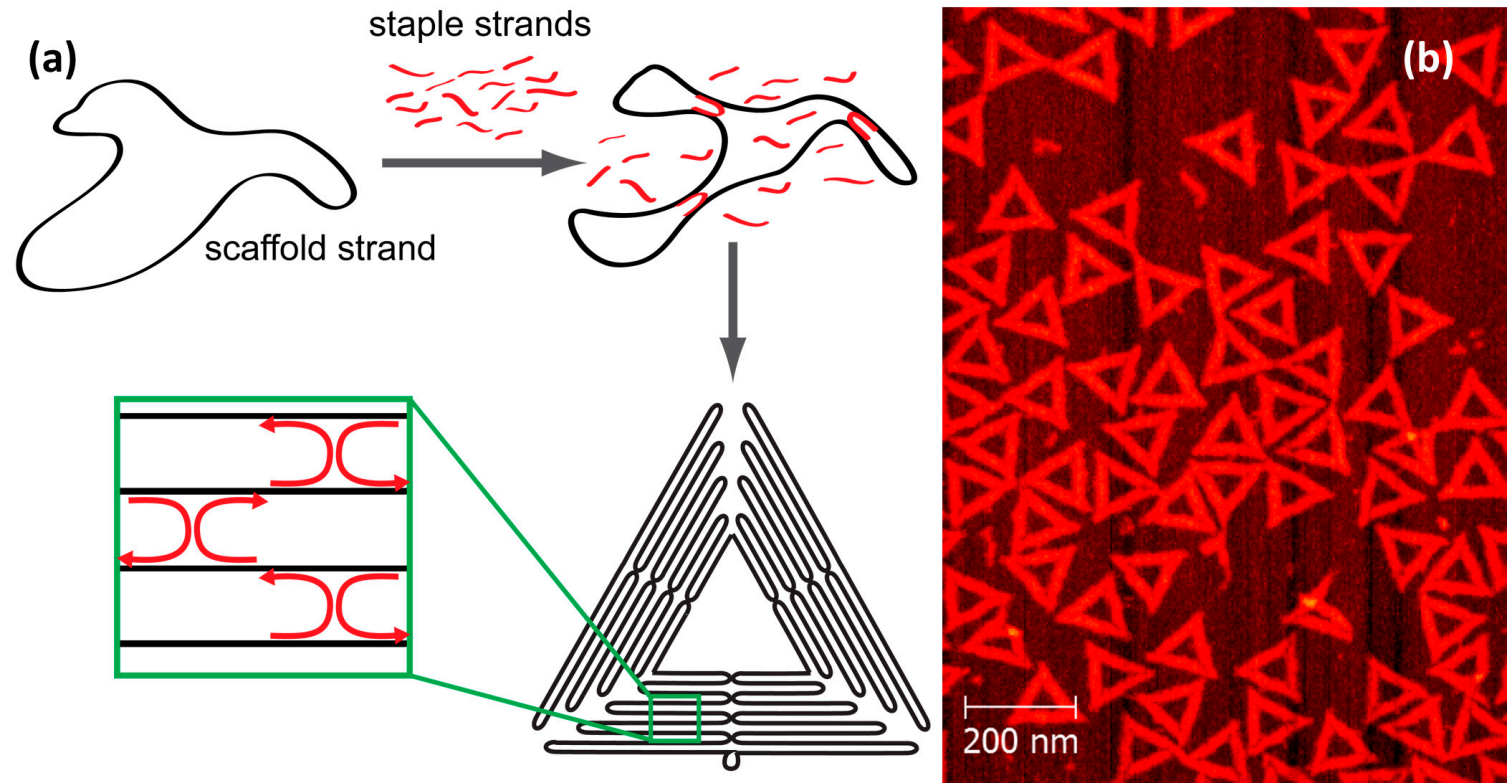
(**a**) Scheme for synthesizing a triangular DNA origami nanostructure: A circular single-stranded scaffold is folded into a triangular shape by addition of ~200 specifically binding oligonucleotides, so-called staple strands; (**b**) Atomic force microscopy (AFM) image of the resulting triangular DNA origami immobilized on a mica surface.

In the DNA origami technique, a long, single-stranded (ss) DNA scaffold (typically a viral genome) is folded into a nanoscale shape by hybridization with a number of short synthetic oligonucleotides, so-called staple strands. Each staple strand is partially complementary to different separated segments of the scaffold strand which causes the scaffold to fold upon hybridization. The resulting DNA origami then consists completely of double-stranded (ds) DNA which is held together by periodic crossovers of the staple strands. The shape of the DNA origami is “programmed” by the sequences of the individual staple strands. Figure 1a shows schematically how a circular DNA strand assembles into a triangular shape upon addition of specific staple strands.

DNA origami assembly is typically performed with a high excess of staple strands in Mg^2+^-containing buffer which screens the electrostatic repulsion between the negatively charged DNA strands. The solution is rapidly heated above the melting temperature of the DNA, *i.e.*, to 60–90 °C, and slowly cooled down to room temperature. During cooling down, the individual staple strands have enough time to find their complementary sequences on the scaffold and fold it into the desired shape.

**Figure 2:**
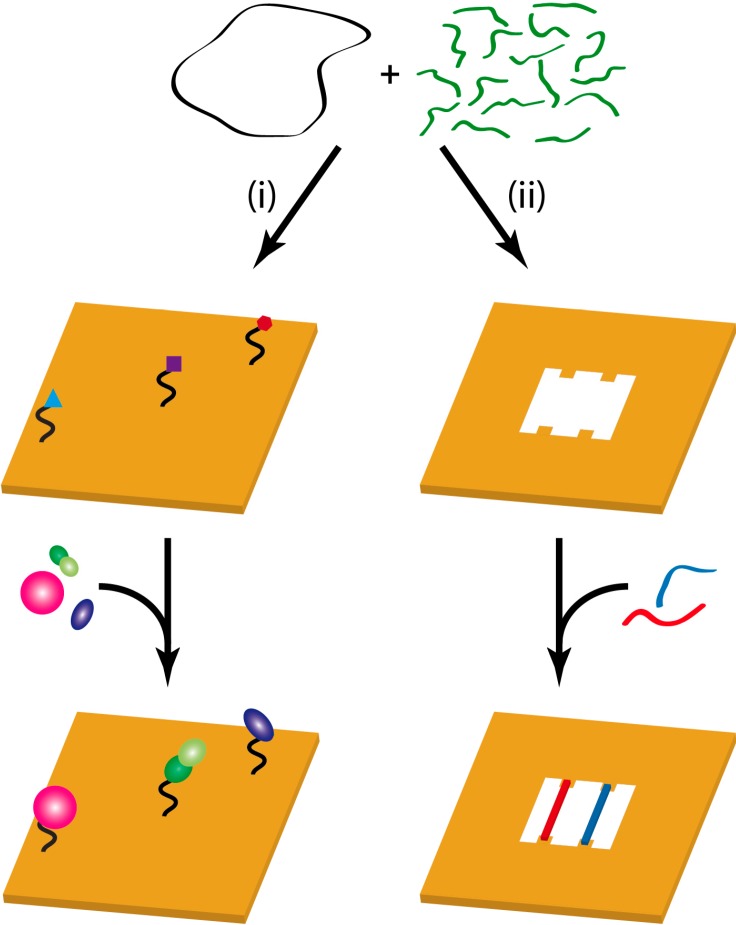
DNA origami nanostructures as substrates for the study of chemical reactions. In strategy (i), a DNA origami is used as a molecular breadboard to arrange molecular entities with high lateral precision while in strategy (ii), a DNA origami frame is constructed for the incorporation of DNA strands of well-defined structure and topology which can then serve as substrates for biochemical reactions.

Most 2D DNA origami consist of the M13mp18 viral DNA scaffold and around 200 staple strands [3]. Each staple strand can be extended to protrude from the DNA origami surface, which results in more than 200 unique sites that can be modified systematically with respect to length, nucleobase sequence, and hybridization state, and synthesized to carry various chemical modifications. Therefore, DNA origami templates are frequently used as locally addressable supports (so-called “molecular breadboards”, see Figure 2 left) for the precise arrangement of functional entities such as plasmonic nanoparticles [4,5], quantum dots [6,7], fluorophores [8,9], and proteins [10,11], which enables their use as templates for the study of chemical reactions at a single-molecule level. For this purpose, also DNA origami frames can be employed (see Figure 2, right) which facilitate the incorporation of single DNA strands with high structural control as substrates for biochemical reactions.

The shapes of DNA origami nanostructures are typically analyzed using single-molecule microscopy techniques [12], the most important being atomic force microscopy (AFM) and transmission electron microscopy (TEM). TEM can yield high-resolution images of single nanostructures, but requires sophisticated sample preparation and image analysis. AFM has the advantage that it can be operated under atmospheric conditions and even in liquids, and the requirements for sample preparation are minimal.

In AFM a cantilever with a sharp tip (radius of apex curvature 1–10 nm) is scanned over the surface and the interaction forces (long range attractive van-der-Waals forces, electrostatic interactions and the shorter range Pauli repulsion) between tip and sample lead to a deflection of the cantilever (in contact mode) or to a reduced vibrational amplitude (in dynamic mode). As a result the topography of a sample’s surface is recorded with lateral resolution of 1–10 nm and height resolution down to 0.1 nm. Under ultra-high vacuum single molecules can be imaged with atomic resolution and even chemical reactions between individual molecules can be observed [13]. Under atmospheric conditions AFM is typically used to image (bio)macromolecules such as DNA and proteins, and due to the accurate height information, the volume of single macromolecules can also be determined. Apart from the topographical information other materials properties can be probed by AFM that are based on the interaction forces, in particular mechanical properties and surface charges.

The typical scan speed of AFM is in the range of several minutes per frame. However, recent developments in high-speed AFM (HSAFM) enable the study of dynamic processes at the sub-second time-scale with scan speeds up to 33 frames per second using small cantilevers with high resonance frequency (≈MHz) and stiff and compact piezoscanners [14].

Other analytical tools that have been used to analyze functionalized DNA origami structures are based on optical methods and can also be operated down to a single-molecule level. Fluorescence spectroscopy is used to detect single molecules and to study, for instance, energy transfer pathways [15,16]. Other optical spectroscopy techniques are based on light scattering, most importantly surface-enhanced Raman scattering (SERS), which was recently demonstrated on a few-molecule level with gold-nanoparticle functionalized DNA origami structures [17,18,19,20]. Single-molecule binding events have also been detected by measuring mechanical transitions in DNA origami using optical tweezers [21]. This review focuses on the application of AFM to study molecular processes on DNA origami nanostructures.

**Figure 3:**
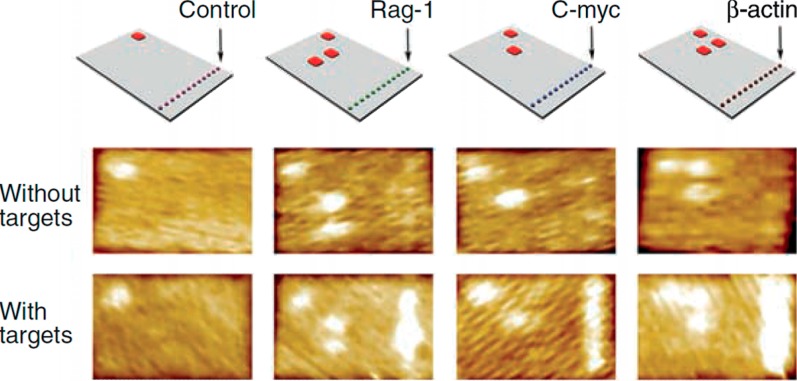
RNA detection using bar-coded DNA origami tiles and AFM analysis. The probe molecules are aligned in a row on the right side of the tiles, and different probes are distinguished from each other with the bar-code located on the top left corner of the tiles. Hybridization is visualized with AFM due to the higher stiffness of double-stranded DNA compared to single-stranded DNA. From Ref. [22]. Reprinted with permission from AAAS.

Soon after the invention of the DNA origami technique by Paul Rothemund in 2006 it was demonstrated that DNA origami tiles can be used as the molecular analogues of macroscopic DNA chips [22]. Rectangular DNA origami structures have been decorated with V shaped pairs of 20 nt long ssDNA and the hybridization with the complementary 40 nt long RNA target sequences was detected by AFM (Figure 3). Twelve binding sites for one target sequence have been placed on each probe tile and the detection was based on the increased stiffness of dsDNA compared to ssDNA. It was estimated that a detection limit of 1000 RNA molecules could be reached if a sample volume of 1 nL was used [22]. To detect RNA with a DNA origami and AFM based approach a similar strategy was recently demonstrated using strand displacement reactions and streptavidin (SAv)-quantum dot reporters [23].

This example shows the potential of DNA origami nanostructures to place molecular binding sites with nanometer precision and to study binding events and molecular processes on a nanometer level by AFM.

## 2. Chemical Reactions

The presence of dsDNA of a certain length (e.g., 40 nt as in the example discussed above) and hairpin loops of a certain size attached to a DNA origami platform can directly be visualized by AFM. Smaller molecules and soft materials are more difficult to observe directly with AFM. Hence, chemical reactions between single molecules can be followed by AFM by visualizing the presence of certain functional groups using SAv as a marker. SAv binds strongly to biotin (Bt) and with its diameter of 5 nm can be easily identified in AFM images. For a detailed discussion of the Bt-SAv interaction, see Section 3.2.

### 2.1. Single-Molecule Chemical Reactions

By using SAv-Bt binding the cleavage of single chemical bonds could be visualized on a single-molecule level. Voigt *et al.* placed three different types of linkers on a rectangular DNA origami platform by extending selected staple strands [24]. It was demonstrated that subsequently the disulfide containing linker can be cleaved with a reducing agent, and a linker containing an electron-rich double bond could be cleaved by using photo generated singlet oxygen. Each cleavage step was followed by observing the disappearing SAv markers at the respective positions of the linkers on the DNA origami rectangle. In the same study the formation of chemical bonds was demonstrated using the same strategy. Subsequently, a click reaction (formation of a triazole from an alkyne and an azide), formation of an amide from an NHS-ester and an amine followed by another click reaction was realized on different positions of the DNA origami and each step was observed by AFM. The yield of the reactions could be determined by analyzing about 200 DNA origami structures.

**Figure 4:**
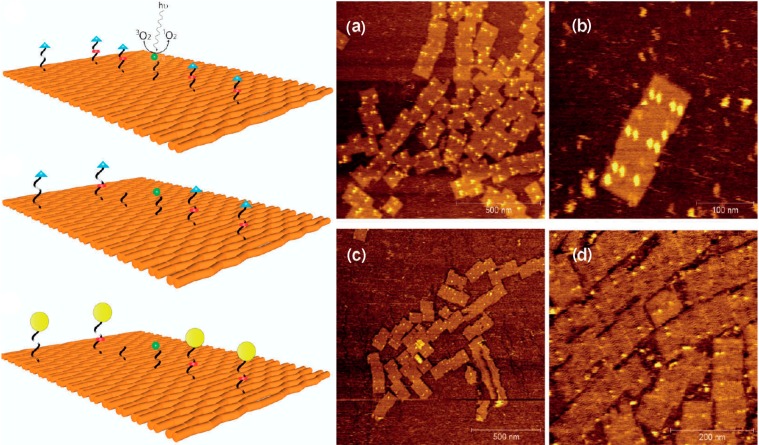
Left: Schematic presentation of the rectangular DNA origami platform used to study singlet oxygen production, diffusion and bond cleavage. Singlet oxygen is produced by an IPS photosensitizer in the center and induces bond cleavage in the SOC linkers placed at different distance from the IPS. After UV-irradiation and thus reaction with singlet oxygen SAv is added, which binds to the remaining strands to visualize them for AFM analyses. On the right examples of AFM images are shown. (**a**) Non-irradiated DNA origami platforms with the SOC linkers and one marker in the corner of the rectangle. A zoom-in is shown in (**b**). UV irradiation results in singlet oxygen production and cleavage of the SOC linkers, which is shown in (**c**,**d**). Adapted with permission from Ref. [25]. Copyright 2010 American Chemical Society.

DNA origami unfolds its real power when the local control of the chemical modifications, *i.e.*, their relative positions, is used to study chemical reactions as a function of distance between the reactants. This was demonstrated by Helmig *et al.* with a photosensitizer placed in the center of a DNA origami rectangle and four singlet oxygen cleavable (SOC) linkers placed at different distances (18 and 36 nm) from the photosensitizer (Figure 4) [25]. Upon UV irradiation singlet oxygen is produced in the center of the DNA origami, which diffuses along the DNA origami. The SOC linkers are cleaved with different yields depending on the distance to the photosensitizer. The reaction was observed on a single-molecule level using AFM and the diffusion controlled linker cleavage induced by singlet oxygen could be analyzed [25].

### 2.2. Electron-Induced Processes

DNA origami platforms have also been used to study reactions of low-energy electrons with DNA oligonucleotides of defined nucleotide sequence [26,27,28]. Electron-induced DNA strand cleavage represents a central elementary reaction in the damage of DNA by high-energy radiation. A detailed knowledge of these processes is beneficial for accurate risk estimates for living organisms exposed to high-energy radiation such as γ- and X-rays and for the improvement of tumor radiation therapy [29,30].

For biological systems DNA strand breaks represent the most important type of radiation damage, since a double strand break can basically not be repaired by enzymes and thus results in cell death. Low-energy electrons (E < 20 eV) are generated in copious amounts along the radiation track of the primary high-energy radiation [31]. It was demonstrated in several studies that low-energy electrons are able to directly induce DNA strand breaks with high cross sections even at energies as low as 0.8 eV by the dissociative electron attachment (DEA) mechanism [32,33]. The determination of electron-induced DNA strand break yields of oligonucleotides of well-defined sequence is a very challenging task, and due to a limited sensitivity of traditional chemical analysis tools such as HPLC only very short oligonucleotides up to a maximum of 4 nt can be analyzed within a reasonable time scale [34]. To study the influence of nucleotide sequence on the electron-induced strand breakage, novel experimental approaches have to be developed and an important step was done by using DNA origami structures as platforms for DNA nanoarrays, whose radiation damage can be studied by AFM [26,27].

**Figure 5:**
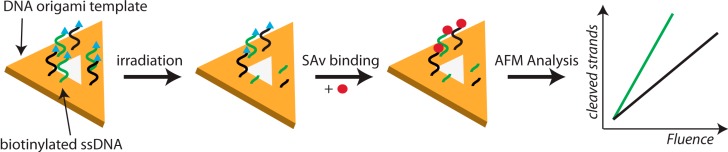
Scheme of the experimental sequence used to quantify electron-induced strand breakages [26]. Triangular DNA origami nanostructures with six protruding strands with two different target structures (indicated in green and black) are irradiated with low-energy electrons.

The experiments are based on a similar procedure as the single-molecule studies of chemical reactions by Voigt *et al.* [24] and Helmig *et al.* [25] described above. SAv binding to biotinylated oligonucleotides protruding from the DNA origami platform is used to visualize intact target strands. The system consisting of target oligonucleotides placed at specific positions on the DNA origami platform is in the following referred to as DNA nanoarray. After irradiation of the DNA nanoarrays in ultrahigh vacuum (UHV) with low-energy electrons DNA strand breaks might occur in the target sequences. The remaining intact strands can be visualized by AFM after addition of SAv. An analysis of a sufficient number of DNA origami structures yields the relative number of strand breaks (N_SB_), and from the slope of the linear regime of the fluence dependence of N_SB_ the yields of DNA strand cleavage at a specific electron energy can be determined. The experimental procedure is schematically shown in Figure 5. The DNA origami technique has several advantages compared to other experimental approaches: (i) Since the detection of DNA strand breaks at a single-molecule level allows for maximum sensitivity, only miniscule amounts of material, corresponding to sub-monolayer coverage, are required; (ii) Two or more different oligonucleotide sequences can be directly compared within one irradiation experiment, which allows for efficient parallel investigation of a number of different DNA structures; (iii) Absolute strand break cross sections are readily accessible due to the single-molecule technique; (iv) The DNA nanoarray technique can be extended to quantify double strand breaks and to investigate higher-order DNA structures. Furthermore, the technique can be applied to other radiation sources such as UV radiation.

**Figure 6:**
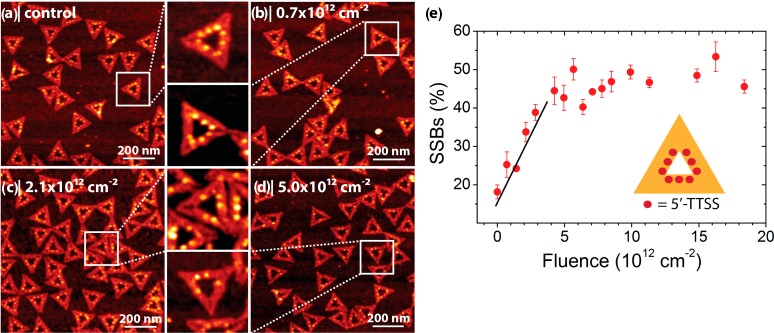
Irradiation of triangular DNA origami structures carrying nine disulfide-containing strands with 18 eV electrons [26]. (**a**) AFM image of a non-irradiated control sample. The sequence of the protruding strands is 5'-TTSS. The DNA origami structures were immobilized on silicon and exposed to SAv, which had a binding efficiency of 80%–90%; (**b**–**d**) AFM images of samples irradiated with different fluences of 18 eV electrons. Due to electron-induced bond cleavage in the protruding strands the number of specifically bound SAv decreases with electron fluence; (**e**) Plot of the relative number of strand breaks (in %) *vs.* electron fluence. The DNA origami design is shown in the inset of (e).

In a proof-of-principle experiment a triangular DNA origami platform was decorated with nine protruding strands containing a disulfide bond, two T nucleotides and the Bt modification: 5'-TTSS [26]. The disulfide bond is very sensitive to electron-induced bond cleavage and was used as a test system. Figure 6 shows examples of AFM images from control samples (non-irradiated) and samples irradiated with different fluences of 18 eV electrons. Figure 6e shows that the number of strand breaks increases linearly with the fluence until a saturation level is reached. From the slope of the linear fit in the low-fluence regime a strand break yield of 1.0 × 10^−3^ was determined [26].

The strand break yields of two different target sequences can be determined in the same irradiation experiment by placing two different sequences on the DNA origami target and arranging them in an asymmetric pattern to make them distinguishable in the AFM images. In this way, the strand break yield for the 5'-TTSS sequence could be directly compared with a 5'-TT sequence without disulfide bond [26]. The damage of the Bt label has to be taken into account as well and in summary the damage yields for 18 eV electron induced damage to the different sequences were found to be: 7.1 × 10^−14^ cm^2^ (5'-TTSS), 1.7 × 10^−14^ cm^2^ (5'-TT) and 1.1 × 10^−14^ cm^2^ (Bt) [27]. Very recently, it was demonstrated that the strand break yields determined with the DNA nanoarray method correspond directly to the absolute cross sections for DNA strand breakage [28].

## 3. Protein Binding Reactions

A variety of methods for the site-specific immobilization of different protein species on DNA origami nanostructures have been reported. Approaches used so far include aptamer-protein binding [35,36], chelate complex formation with His-tagged proteins [37], the sequence-specific binding of zinc-finger proteins to dsDNA [11], and most prominently SAv-Bt binding [10,38,39,40,41]. Recently, also the growth and arrangement of amyloid fibrils on DNA origami templates has been demonstrated [42]. Due to the comparatively large size of most proteins, AFM provides a powerful tool for the identification of individual proteins arranged in complex arrays on DNA origami substrates. Consequently, AFM of functionalized DNA origami has been employed at several instances to monitor and quantify protein binding dynamics and selectivity.

### 3.1. Aptamer-Protein Binding

Aptamers are short ssDNA sequences which recognize and bind specific protein sites via their 3D structure by wrapping around the target molecule [43]. They can be easily arranged on DNA origami nanostructures by extending selected staple strands with the desired aptamer sequence [35]. In this way, Rinker *et al.* exploited the spatial addressability of DNA origami to arrange two different aptamers into parallel lines with controlled distance [36]. The different aptamer sequences recognized two opposite sites of the blood coagulation protein thrombin which has a diameter of about 4 nm. By varying the distance between the aptamers, AFM revealed that binding occurred selectively at an aptamer-aptamer distance of 5.3 nm while almost no binding was observed for a distance of 20.7 nm. In these experiments, the rigidity of the DNA origami substrate plays a key role as it minimizes spatial fluctuations in the aptamer-aptamer distance and thus enhances binding selectivity.

A similar DNA origami design has recently been used to detect the DNA repair activity of human O^6^-alkylguanine-DNA alkyltransferase (hAGT) [44]. To this end, DNA origami were again decorated with parallel lines of the two thrombin-binding aptamers. Introduction of one methylated guanine into one of the aptamers disrupted the 3D structure of the aptamer and thus suppressed thrombin binding. The methylated guanine, however, could be repaired by incubation with hAGT which fully restored the thrombin-binding activity of the aptamer.

### 3.2. Streptavidin-Biotin Binding

The binding of SAv to Bt is among the strongest non-covalent interactions, exhibiting a dissociation constant *K_d_* of 10^−14^ M–10^−15^ M [45]. SAv has a spherical shape and consists of four identical subunits, each of which can bind one Bt molecule. Due to its size of ~5 nm in diameter and the well-established modification of oligonucleotides with Bt, SAv is widely used as a marker to visualize individual sites on DNA origami by AFM (see Section 2). Furthermore, it has been demonstrated that biotinylated ssDNA strands of sufficient length can “thread” through the holes in the 2D DNA origami sheet, enabling efficient SAv binding also for DNA origami substrates that are adsorbed in a face-down configuration, *i.e.*, with the Bt modifications facing toward the solid surface and away from solution [26,46].

The dynamics of SAv-Bt binding on DNA origami substrates was studied at a single-molecule level by Wu *et al*. [40]. After immobilization of DNA origami with four Bt modifications on mica, the authors exposed the substrates to a constant concentration of SAv and monitored the amount of specifically bound SAv on the DNA origami surfaces over time by time-lapse AFM. Over a course of 30 min, the ratio of bound SAv per available Bt site slowly increased until saturation was reached at close to 100%. By repeatedly scanning the same area, it was further shown that the SAv-Bt binding is strong enough not to be affected by the forces exerted by the AFM tip.

**Figure 7:**
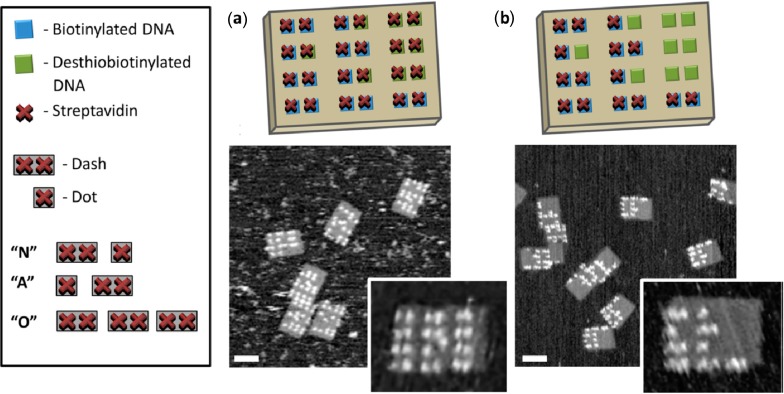
Design of the SAv Morse code arrangement on DNA origami substrates with corresponding code translation. After SAv binding, the DNA origami display the Morse code for “OOOO” (**a**). After addition of free Bt, the selective release of SAv from the origami results in the message “NANO” (**b**). Reprinted with permission from Ref. [41]. Copyright 2013 American Chemical Society.

Reversible SAv immobilization on DNA origami was demonstrated by Wong *et al.* [41]. To this end, 2D DNA origami nanostructures carrying both Bt and desthiobiotin (dBt) modifications were employed. Similar in its structure to Bt, dBt is also able to bind SAv but with a much higher dissociation constant (*K_d_* ~ 10^−11^ M). Therefore, upon exposure to the DNA origami, SAv was binding to both modifications which resulted in the appearance of a pattern associated with the Morse code “OOOO” as observed by AFM (see Figure 7a). The binding yield of SAv to Bt was found to be ~95% while SAv-dBt binding resulted in a yield of only ~84%, reflecting the higher dissociation constant. The authors then added free Bt to the SAv-decorated DNA origami. In the case of the Bt-immobilized SAv, free Bt was captured by the remaining three unoccupied SAv subunits but the protein remained at its predefined binding site on the origami surface. For SAv bound to dBt, however, free Bt displaced dBt due to its higher binding affinity, thus releasing the immobilized SAv from the DNA origami. In the AFM images, the selective release could be observed by the appearance of the Morse code for “NANO” (see Figure 7b). The authors furthermore demonstrated that the original message can be restored after washing away the free Bt by a second exposure to SAv, which then binds to the newly unmasked dBt sites.

SAv can be immobilized on a variety of inorganic species such as gold nanoparticles [47] or quantum dots (QDs) [48] which enables their coupling to biotinylated molecules. By investigating the binding of SAv-coated QDs to different DNA origami designs, Ko *et al.* demonstrated the great potential of AFM in combination with DNA origami substrates not only to visualize but to gain quantitative information about binding reactions [7]. The QDs used in their study had a diameter of ~20 nm and were coated with 5–10 SAv molecules per QD. The authors first studied the effect of monovalent, *i.e.*, one Bt per binding site, *vs.* trivalent binding, *i.e.*, three Bt molecules per binding site. Trivalent binding was found to drastically enhance the binding yield from ~22% to ~90%. By varying the distance between the individual binding sites on the origami surface between 22 and 50 nm, steric hindrance effects in QD binding were assessed. Steric hindrance led both to a reduced binding yield and to poor spatial precision in the QD arrangement when the spacing between binding sites was less than twice the hydrodynamic radius of the QDs. Using AFM, the authors could also quantitatively assess the QD binding kinetics and found that the SAv-QD DNA origami hybrids have a *K_d_* that exceeds that of pure SAv-Bt by up to seven orders of magnitude. Increasing the linker length, *i.e.*, the length of the biotinylated protruding strands, led to a considerable increase in reaction rate while *K_d_* was barely affected.

### 3.3. DNA-Binding Proteins

Many DNA-binding proteins induce distortions in DNA upon binding which requires a certain flexibility of the DNA strand. DNA origami substrates provide the unique opportunity to immobilize target DNA strands for protein binding and control their structural properties in a well-defined manner.

**Figure 8:**
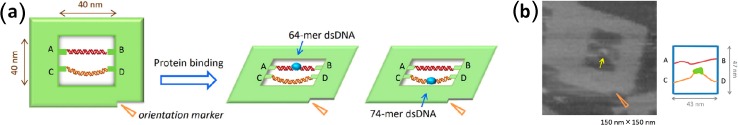
(**a**) Scheme of the DNA origami frame with a tensed 64-mer (red) and a relaxed 74-mer DNA substrate (orange) for Sox2 binding; (**b**) AFM image of Sox2 bound to the relaxed substrate strand (**left**) and representation of the kinked geometry of the substrate strand obtained from the AFM image (**right**). Adapted with permission from Ref. [49]. Copyright 2014 American Chemical Society.

Yamamoto *et al.* recently used this approach to study the cooperative binding of the transcription factors Sox2 and Pax6 to the DC5 element of the δ1-crystallin gene in dependence of DNA tension [49]. To this end, the authors employed a DNA origami frame in which two DNA strands exhibiting the DC5 element were immobilized (see Figure 8a). By controlling the lengths of the strands, the degree of tension could be varied. An orientation marker in the DNA origami frame enabled the unambiguous identification of the individual DNA strands in AFM images while the bound proteins appeared as protrusions on the strands. For the more flexible strand, the authors observed an almost twofold increase in Sox2 binding compared to the shorter, tensed strand. Furthermore, the Sox2 binding to the flexible strand was often accompanied by a kink in the substrate strand (see Figure 8b) which indicates that although Sox2 can bind to stiff DNA, the binding affinity is reduced as bending of the substrate is prohibited. In retinal tissues, complexes of Sox2 and Pax6 are involved in the expression of the lens protein δ-crystallin. Although Pax6 alone did not bind to either the tensed or the relaxed strand, cooperative binding was observed in the presence of both proteins with the Sox2-Pax6 complex being characterized by an increased volume compared to Sox2 alone. In addition, it was observed that the presence of Pax6 almost doubled the binding yield to the relaxed DNA strand while it resulted only in a marginally increased binding to the tensed strand. These results show that the cooperativity depends on the initial binding and bending of the DNA strand by Sox2 which makes it accessible for Pax6 and leads to a further increase in Sox2-Pax6 complex formation on the DNA strand.

## 4. Enzymatic Reactions

Enzymes are proteins that are able of catalyzing biochemical reactions. By decorating DNA nanostructures with enzyme cascades or multi-enzyme complexes in different configurations, such reactions can be studied in dependence of various parameters including enzyme distance, arrangement, and stoichiometry [50,51]. Many biological processes such as DNA replication, transcription, and repair, however, involve the direct and highly specific interaction of enzymes with target DNA sequences, with enzyme activity often being influenced by the target DNA’s structural properties. Immobilizing target DNA sequences on DNA origami substrates and controlling their structural properties therefore enables the investigation of DNA-binding enzymes under well-defined conditions at a single-molecule level. In particular, HSAFM of such DNA origami substrates allows for the real-time study of enzyme binding, diffusion, and activity. For instance, by attaching a long dsDNA featuring a T7 promoter region to a rigid DNA origami substrate, the movement of a T7 RNA polymerase along the dsDNA and the resulting transcription product could be directly observed by HSAFM [52].

Endo *et al.* have used the DNA origami frame discussed in Section 3.3. to study the activity of various enzymes in dependence of DNA tension. Their first experiments focused for instance on the DNA methylation enzyme M.*Eco*RI which specifically recognizes the sequence GAATTC [53]. They found, that the yields of M.*Eco*RI binding varied significantly for the two different strands: a binding yield of 87% was observed for the relaxed strand while the binding yield for the tensed strand was only 13%. This demonstrates the importance of structural alterations induced by M.*Eco*RI upon binding. In line with this observation, also diffusion of the enzyme along the DNA strand was inhibited on the tensed strands. Finally, the activity of M.*Eco*RI was investigated in dependence of DNA tension by using a second enzyme, R.*Eco*RI, which was able to cleave both the tensed and relaxed non-methylated strands. Methylation, however, inhibits cleavage by R.*Eco*RI. Therefore, the authors investigated the yield of R.*Eco*RI cleavage after treatment with M.*Eco*RI for the two strands and found that 87% of the tensed strands were cleaved but only 57% of the relaxed strands. This demonstrates that DNA tension hinders DNA methylation by M.*Eco*RI.

The same approach was also used to study the activity of the base excision repair enzymes hOgg1 and PDG which selectively remove 8-oxoguanine and pyrimidine dimers, respectively, by introducing these sites into the target strands opposite to a nick [54]. Base excision thus results in a double strand break which can be identified in the AFM images. For both enzymes, base excision was suppressed for the tensed DNA strands, *i.e.*, 11% *vs.* 32% for hOgg1 and 7% *vs.* 29% for PDG. The authors were furthermore able to directly visualize the individual reaction steps of PDG base excision, *i.e.*, binding to the target strand, diffusion along the strand, strand cleavage, and PDG dissociation, in real time by HSAFM.

**Figure 9:**
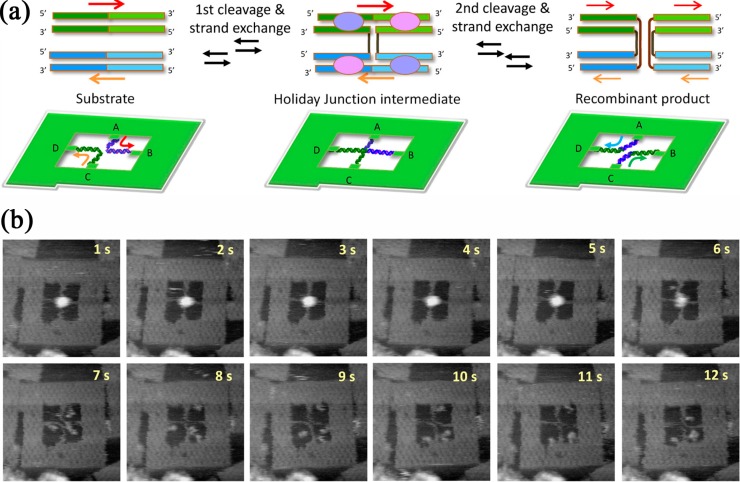
(**a**) Scheme of the DNA origami frame with a crossed geometry of attachment sites for the immobilization of *loxP*-containing DNA strands in antiparallel configuration and pathway of Cre-*loxP* recombination via an intermediate Holliday junction; (**b**) HSAFM images of the reaction, starting with the synaptic complex. Adapted with permission from Ref. [55]. Copyright 2013 American Chemical Society.

Using a modified DNA origami frame with a crossed geometry of attachment sites for the target strands, Suzuki *et al.* investigated the site-specific recombination between two *loxP* sites by the *E.coli* phage P1 Cre recombinase with HSAFM [55]. In particular, the authors immobilized two DNA strands containing *loxP* sites across the edges of the DNA frame (Figure 9a, left) either in parallel or antiparallel configuration. Upon exposure to Cre, four Cre monomers were binding to the two strands and forming a synaptic tetramer complex. The first strand exchange then resulted in an intermediate Holliday junction (Figure 9a, center), while the second strand exchange yielded the recombinant products that had different connection patterns and were therefore distinguishable from the substrates (Figure 9a, right). The time course of the reaction as monitored by HSAFM is shown in Figure 9b for the antiparallel configuration. The synaptic complex, its disintegration into four monomers, and the recombinant products can be clearly seen. For the parallel configuration of *loxP* sites, a synaptic complex was observed but no recombination. From these experiments, the authors concluded that antiparallel *loxP* sites can be converted from the substrates to recombinant products through the formation of synaptic complexes while parallel sites do not recombine efficiently even though they can be synapsed. In further experiments using Holliday junctions as starting substrates tethered to two different DNA origami frames, the authors could also establish a relation between the preferential cleavage site of Cre in the *loxP* sequence and the topological state of the Holliday junction which again demonstrated the importance of substrate bending by the enzyme for the reaction. Using a similar approach, also the influence of Holliday junction flexibility on the activity of the RecU Holliday junction resolvase was investigated [56]. It was found, that although the resolvase could bind both to flexible and inflexible Holliday junctions, flexible junctions were resolved more efficiently.

## 5. Conformational Transitions of DNA

### 5.1. Guanine Quadruplexes

G quadruplexes can be formed from telomeric DNA, which is a single stranded G rich sequence present at the ends of eukaryotic chromosomes. Telomeric DNA protects and stabilizes the genome, and its length determines the lifetime of a cell [57]. In mammals the telomeric DNA consists of the repeat unit 5'-(TTAGGG)_n_ [58]. In the presence of monovalent cations four G bases form a tetrad through intermolecular Hoogsteen-type hydrogen bonding, and two or three G tetrads can be stacked on top of each other stabilized by π-π interactions and coordination with central cations. The formation of single G quadruplexes upon addition of K^+^ ions was directly observed by AFM using the DNA origami frame introduced above [59]. From two parallel DNA duplexes spanning two sides of the frame single stranded extensions were introduced containing the G tracts 5'-GGGTTAGGGTTAGGGTTT and 5'-TTTGGGT, respectively. After addition of K^+^ an intermolecular G quadruplex is formed resulting in a transition from the parallel DNA duplex to an X shape, which can be directly observed by AFM.

**Figure 10:**
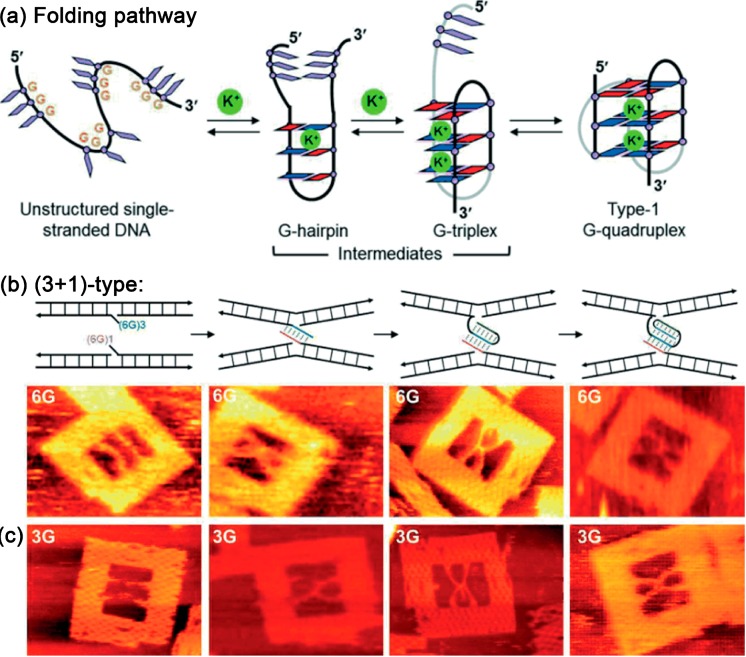
(**a**) Proposed folding pathway of the human telomeric type-1 G-quadruplex including the intermediates G hairpin and G triplex; (**b**) Scheme of different strand designs within the DNA origami frame that are able to form the G hairpin, G triplex, or a G quadruplex in presence of K^+^. Corresponding AFM images of the different designs show that without K^+^, but a (3+1)-type (6G in (b) and 3G in (**c**)) design no G-quadruplexes are formed (parallel arrangement). In the presence of K^+^ and using DNA strands capable of forming G hairpins, G triplexes, or G quadruplexes, respectively, the X shapes are observed. Adapted from Ref. [60]. Copyright Wiley VCH Verlag GmbH & Co KGaA. Reproduced with permission.

In a very recent study it was demonstrated that the formation of the G quadruplex might proceed via two transition states, a G hairpin (*i.e.*, a G-G mismatched Hoogsteen duplex), and a G triplex (*i.e.*, a partly folded structure with three tandem G repeats bound by Hoogsteen hydrogen bonding, see Figure 10a) [60]. The formation of the transition states was demonstrated with two types of structures, a tetramolecular antiparallel and a (3+1)-type G quadruplex structure. It was found that the G-hairpin structure forms also in presence of Mg^2+^ ions (with a yield of 64%) and the yield did not increase notably when adding K^+^ ions indicating that the G hairpin selectively prefers Mg^2+^ over K^+^ ions. The G triplex structure was formed with considerable yield in the presence of both Mg^2+^ and K^+^ ions (43% and 54%, respectively), whereas the highest yield of the G quadruplex structure was obtained only in presence of K^+^ ions (76%). This study suggests that the intermediate states are preferentially formed when Mg^2+^ is present, and the transition to the G quadruplex is most efficient with K^+^.

G quadruplexes are also of considerable medical relevance. In most cancer cells the enzyme telomerase is overexpressed by maintaining and extending the telomere sequences thereby extending the life span of cells. In the context of cancer therapy numerous quadruplex-binding ligands have been identified [61]. The DNA origami based single-molecule analysis of G quadruplex-ligand binding has very recently been demonstrated to be very attractive, since the G quadruplex structure (based on number of involved strands, strand polarity and sequence) can be effectively controlled on the single-molecule level, which is not the case for bulk solutions that usually consist of a complex mixture of different G quadruplex structures [62]. In this way the binding of a pyrido-dicarboxamide (PDC) ligand modified with Bt to four G tracts was studied. The G tracts were incorporated into two G-G mismatched duplexes attached to the DNA frame. Binding of the PDC ligand induced G quadruplex formation, which was again visualized by AFM via the X shape of the interlinked duplexes in the center of the DNA frame. The presence of the PDC linker was further confirmed by the addition of SAv, which bound to the Bt modification of PDC thereby visualizing the position of PDC. A reversible PDC-induced G quadruplex formation and unbinding was observed by HSAFM most likely representing formation of intermediate states before a stable final configuration was adopted. The HSAFM measurements also revealed that PDC-induced G quadruplex formation is considerably slower than K^+^ induced binding [62].

With the same technique also the nucleocapsid protein (NCp) of the human deficiency virus type 1 (HIV-1) was studied [63]. G-rich sequences were shown to inhibit HIV-1 replication [64]. HIV-1 NCp is a multifunctional protein that promotes and stabilizes G quadruplex structures. By using the DNA origami frame and HSAFM the real-time NCp induced G quadruplex formation was imaged and translocation events (1-D sliding, 3-D hopping, loop formation) were captured as part of the NCp-telomer searching mechanism [63].

**Figure 11:**
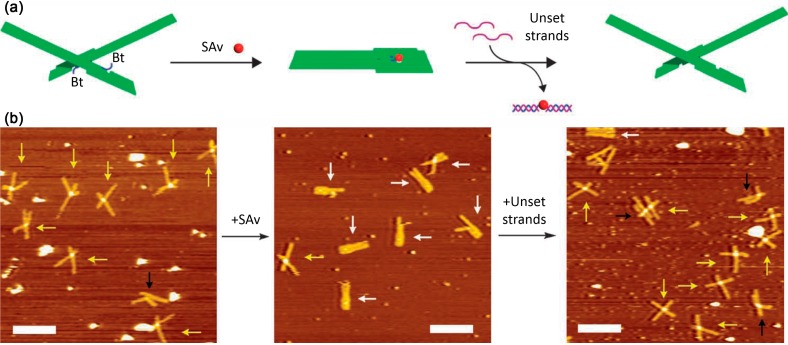
(**a**) Shape transition of the nanomechanical DNA origami device upon SAv binding and release; (**b**) Corresponding AFM images. Adapted by permission from Macmillan Publishers Ltd: Ref. [38], copyright 2011.

### 5.2. Nanomechanical DNA Origami Devices for Molecular Detection

Kuzuya *et al.* designed a nanomechanical DNA origami device which undergoes a shape transition upon target binding and release [38]. The device consists of two symmetric levers attached to each other via an immobile Holliday junction to resemble a plier geometry (see Figure 11a). Each of the levers further featured a single Bt modification. When adsorbed to mica surfaces, AFM revealed that the adsorbed devices predominantly had a cross geometry (see Figure 11b, left). In the presence of SAv, the Bt modifications of each lever could bind to two different subunits of one SAv molecule which led to a shape transition of the device. In the AFM images, the SAv-bound devices predominantly displayed a parallel geometry (see Figure 11b, center). The Bt modifications, however, were not attached directly to staple strands. Instead, biotinylated oligonucleotides were hybridized to protruding strands so that the Bt modifications and therefore the captured SAv molecules could be released again via a strand displacement reaction which reversed the shape transition (see Figure 11b, right). The versatility of the nanomechanical DNA origami devices was further demonstrated by employing a number of different capture and release mechanisms to detect a variety of molecular species using the same shape transition. In particular, anti-fluorescein IgG was detected via FAM modifications, Na^+^ and K^+^ via G quadruplex formation, Ag^+^ via C-C mismatch stabilization, miRNA via strand displacement reactions, and ATP via aptamer modifications.

## 6. Conclusions

With the DNA origami technique, well-defined nanostructures of arbitrary shape can be synthesized. Due to the high structural control provided by the technique and the possibility to arrange molecular entities with highest precision, DNA origami have therefore become a promising substrate for investigating molecular processes at a single-molecule level. In combination with AFM as a high-resolution imaging technique, not only the visualization but also the quantitative study of chemical and biochemical reactions becomes possible.

The works discussed in this review have demonstrated the great potential of this approach and incredible advances have been made in past few years. The applications range from the kinetics of protein binding reactions, to molecular detection with highest sensitivity, to enzymatic reactions, to radiation-induced bond cleavage. The progress made in HSAFM imaging has even enabled the real-time study of enzyme binding, diffusion, and activity in dependence of a number of parameters, most importantly DNA tension and structure.

Further advances in the field can be expected in the years to come. The already large number of studies focusing on G quadruplexes may be further extended to other higher order structures such as i-motifs while the quantitative investigation of radiation-induced reactions by AFM on DNA origami substrates is only in its early infancy. The previous studies of electron-induced strand breakage in ssDNA may easily be extended to more complex DNA and even DNA-protein targets, and to other types of radiation such as photons and ions. AFM imaging of DNA origami nanostructures is thus likely to develop into a well-established technique for the single-molecule investigation of a large number of molecular processes and reactions.
